# Surrogate markers of adiposity and elevated blood pressure in African children

**DOI:** 10.11604/pamj.2023.46.114.42371

**Published:** 2023-12-22

**Authors:** Aude Laetitia Ndoadoumgue, Ulrich Flore Nyaga, Jean Jacques Noubiap

**Affiliations:** 1 School of Health and Related Research, The University of Sheffield, Sheffield, United Kingdom,; 2 Health Data Acumen, Nairobi, Kenya,; 3 Department of Medicine, University of California-San Francisco, San Francisco, California, USA

**Keywords:** Hypertension, obesity, children

## Editorial

Hypertension is one of the leading global health problems due to its huge associated morbidity and mortality. According to the Global Burden of Disease study in 2019, elevated blood pressure (BP) caused 235 million disability-adjusted life years (9.3% of total disability-adjusted life years) and 10.8 million deaths (19.2% of total deaths) globally [1]. Hypertension has traditionally been tied mainly to adults. Although hypertension has long been overlooked in children and adolescents, there is growing evidence that hypertension is prevalent in children and adolescents [2]. There are significant variations in the estimates of the prevalence of primary hypertension in childhood, depending on the measurement techniques used, definition of elevated BP, and setting (location, population). However, it is estimated that the prevalence of elevated BP in the general pediatric population varies between 13% and 18% and that of hypertension ranges from 2% to 5% [2]. It is now well-established that elevated BP in childhood and adolescent tracks into adulthood and has detrimental lifelong cardiovascular effects [2,3]. It is estimated that in about half of cases, hypertension in adults started during childhood [4]. Furthermore, individuals with elevated BP who have higher BP trajectories or from the middle of childhood have evidence for target organ injury as young adults [2,3]. Indeed, high BP in childhood or adolescence has been associated with high carotid India-media thickness, high pulse wave velocity, and left ventricular hypertrophy in adulthood [5]. Elevated BP in children has also been linked with mortality in adulthood and cardiovascular disease [5].

Excess adiposity resulting from unhealthy diet and reduced physical activity is a major driver of hypertension in children and adolescents. Similar to adults, an excess of body fat increases the risk of primary hypertension in children. Analysis of NHANES data spanning from 2015 to 2018 revealed that children who are obese were more prone to hypertension compared to those with a normal weight [2]. Several pediatric studies examining body mass index (BMI) and other measures of adiposity consistently demonstrate a positive link between hypertension and obesity [2]. Childhood obesity is a serious public health problem with an alarming increase in prevalence worldwide [6]. The highest prevalence of childhood obesity has been observed in high-income countries [6]. Whereas this prevalence has stabilised at high levels in most of these high-income countries, there is an increasing trend in low and middle-income countries [7]. Overall, the age-standardized prevalence of obesity in children and adolescents aged 5-19 years rose from 0.7% to 5.6% for girls and from 0.9% to 7.8% for boys from 1975 to 2016 [8]. The World Obesity Federation projected that by 2025, approximately 206 million children and adolescents aged 5-19 years would be obese, and this number is anticipated to rise to 254 million by 2030 [9]. Approximately 55% of children living with obesity go on to become obese adolescents, around 80% of these adolescents living with obesity will remain obese in adulthood and approximately 70% will remain obese over age 30 years [10]. This will ultimately lead to an increased risk of adverse health outcomes such as diabetes, metabolic disorders, fatty liver disease, and obesity-associated complications such as coronary heart disease, asthma, and obstructive sleep apnoea syndrome disease [7].

In this issue of the Pan African Medical Journal, Niba *et al*. report a study on the relationship between elevated BP and surrogates of adiposity in secondary school adolescents in an urban setting in Cameroon [11]. The study aimed to determine the proportion of secondary school adolescents with hypertension or elevated BP with respect to some measures of adiposity (waist circumference, waist-to-height ratio, BMI) and to assess the association between BP and adiposity indices in this population. The overall prevalence of elevated BP and hypertension was 39.2% (with 8.6% and 7.9% of the hypertensive children in stage I and stage II respectively). The prevalence of elevated BP and hypertension was respectively 33.3% and 33.3% in the BMI-obese children, 25.9% and 25.2% in the waist circumference overweight/obese children and 29.4% and 41.2% in the “high risk” (waist-to-height ratio ≥ 0.5) children. Waist circumference overweight/obese, “high risk” children and body mass index-obese had a significantly higher mean systolic blood pressure and diastolic blood pressure compared to their counterparts with normal adiposity indices. Only waist circumference in these children showed a positive, significant and independent relationship with systolic blood pressure [11].

These findings align with the evidence from a meta-analysis and systematic review which showed a prevalence of 12.7% for slightly elevated BP (systolic or diastolic blood pressure ≥ 90^th^ percentile and <95^th^ percentile) and 5.5% for elevated BP (systolic or diastolic blood pressure ≥ 95^th^ percentile) among adolescents and children in Africa [12]. Furthermore, there is also a strong association between overweight/obesity and elevated BP in children and adolescent, across genders, various age groups, and among diverse geographic and ethnic [2,3,12]. Concerning rates of metabolic syndrome have been reported in children and adolescents in Africa [13]. The finding that, among the surrogates of adiposity investigated, only waist circumference had a significant relationship with systolic BP is of paramount importance. Although it needs to be replicated in other populations, it suggests that waist circumference might be a better predictor of hypertension in children and adolescents, compared to BMI for instance, and could be used to identify in this population those at risk of developing hypertension and who might need some interventions including lifestyles modification and BP tracking. The evidence of increasing prevalence rates of elevated BP, hypertension, and obesity in children and adolescents in Africa calls for effective interventions to curb these trends. Such interventions need to be aimed at promoting better eating habits and physical activity. Nutrition and physical activity programs in schools and social marketing campaigns directed to parents have been shown to improve children and adolescent lifestyles [14,15]. Regulations that restrict the consumption of unhealthy food, subsidies for low-income households to support healthy diets, and policies aimed at fostering physical activity, achieved through expanding secure areas for recreational activities and creating environments that support non-motorized transportation, are also important interventions for the primordial cardiovascular prevention in this population [15]. Digital technologies also offer unique opportunities for health promotion in children and adolescents.

The burden of elevated BP and obesity in African children is concerning. Health promotion interventions tailored to this population are highly needed to build a strong foundation for cardiovascular health in later life. Future studies should explore trajectories of BP and adiposity from childhood to adulthood in African populations and determine the impact of elevated BP and overweight/obesity in children on the occurrence of cardiovascular disease in adults. Whether waist circumference is the surrogate of adiposity with the strongest association with BP in children should be further investigated with clear and robust designs.
